# Guest Editorial

**Published:** 2013-12-26

**Authors:** Charles Lekic

## Abstract

International Journal of Clinical Pediatric Dentistry is 5 years old and over this period of time has achieved the reputation of a well-managed clinical journal cited by many authors around the world.

International Journal of Clinical Pediatric Dentistry is 5 years old and over this period of time has achieved the reputation of a well-managed clinical journal cited by many authors around the world. This journal is unique in providing complete, definitive and authoritative reviews on many aspects of clinical pediatric dentistry and continues to be a significant contribution to the art of this speciality. At the same time, we know that there will always be room for improvement. The Editor and the Editorial Board have worked with the outstanding publishing staff to design the journal with a very contemporary and easy to follow outlook. All figures and photographs are of high color quality and could be found in the online version of the manuscript, enabling easy access to a number of very relevant clinical conditions. To further broaden the outreach and appeal of the topics published, we try to emphasize the need for multidisciplinary approach in clinical pediatric dentistry aiming at integrating research in this field with other oral health and general health disciplines. In this way, we hope to cross many bridges that will broaden the impact of the International Journal of Clinical Pediatric Dentistry in both the basic and clinical research domains. It is important to add that the Editorial Board of the journal is international-based which provides a real international outreach. We are certain that the international expertize of our board members will keep the journal up to date in regard to the clinical and scientific significance of the topics discussed. In closing this message I would like to thank the Editor and the Board members for their continued efforts in having the International Journal of Clinical Pediatric Dentistry provide an important contribution to the art and science of pediatric dentistry.


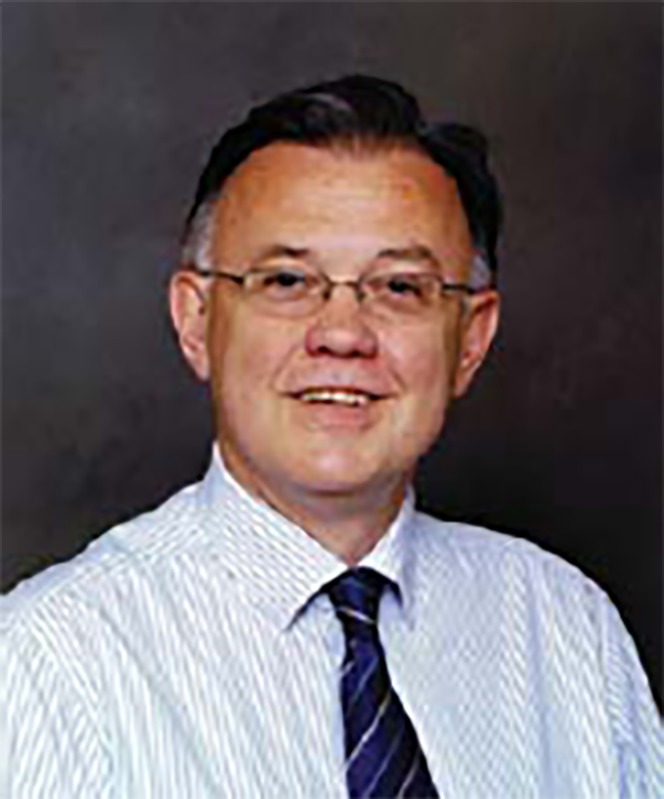
**Charles Lekic**
DDM, MSc, PhD, FRCD(C) *Associate and Managing Editor*

